# Effects of canagliflozin on amputation risk in type 2 diabetes: the CANVAS Program

**DOI:** 10.1007/s00125-019-4839-8

**Published:** 2019-03-12

**Authors:** David R. Matthews, Qiang Li, Vlado Perkovic, Kenneth W. Mahaffey, Dick de Zeeuw, Greg Fulcher, Mehul Desai, William R. Hiatt, Mark Nehler, Elisa Fabbrini, Mary Kavalam, Mary Lee, Bruce Neal

**Affiliations:** 1 Oxford Centre for Diabetes, Endocrinology and Metabolism, University of Oxford, Oxford, UK; 2 Harris Manchester College, University of Oxford, Mansfield Road, Oxford, OX1 3TD UK; 3 The George Institute for Global Health, UNSW Sydney, Sydney, NSW Australia; 4 The Royal North Shore Hospital and University of Sydney, Sydney, NSW Australia; 5 Stanford Center for Clinical Research, Department of Medicine, Stanford University School of Medicine, Stanford, CA USA; 6 University Medical Center Groningen, University of Groningen, Groningen, the Netherlands; 7 Janssen Research & Development, LLC, Raritan, NJ USA; 8 Division of Cardiology and CPC Clinical Research, University of Colorado School of Medicine, Aurora, CO USA; 9 Division of Vascular Surgery and CPC Clinical Research, University of Colorado School of Medicine, Aurora, CO USA; 10 Epidemiology and Biostatistics, Imperial College London, London, UK

**Keywords:** Clinical diabetes, Clinical science, Diabetic foot, Human, Oral pharmacological agents

## Abstract

**Aims/hypothesis:**

The primary analysis of the Canagliflozin cardioVascular Assessment Study (CANVAS) Program showed canagliflozin to have a beneficial effect on cardiovascular and renal outcomes in people with type 2 diabetes at high cardiovascular risk, but also an unexpected increased risk of major or minor lower extremity amputation. These secondary analyses explore this finding in more detail.

**Methods:**

The effect of canagliflozin on amputation risk in the CANVAS Program was calculated for amputations of different types and proximate aetiologies and different canagliflozin doses. Univariate and multivariate associations of baseline characteristics with amputation risk were determined and proportional and absolute effects of canagliflozin were compared across subgroups.

**Results:**

There were 187 (1.8%) participants with atraumatic lower extremity amputations (minor 71%, major 29%); as previously published, rates were 6.30 vs 3.37 per 1000 participant-years with canagliflozin vs placebo (HR 1.97 [95% CI 1.41, 2.75]). Risk was similar for ischaemic and infective aetiologies and for 100 mg and 300 mg doses. Overall amputation risk was strongly associated with baseline history of prior amputation (major or minor) (HR 21.31 [95% CI 15.40, 29.49]) and other established risk factors. No interactions between randomised treatment and participant characteristics explained the effect of canagliflozin on amputation risk. For every clinical subgroup studied, numbers of amputation events projected were smaller than numbers of major adverse cardiovascular events averted.

**Conclusions/interpretation:**

The CANVAS Program demonstrated that canagliflozin increased the risk of amputation (mainly minor) in this study population. Anticipated risk factors for amputation were identified, such as prior history of amputation, peripheral vascular disease and neuropathy, but no specific aetiological mechanism or at-risk subgroup for canagliflozin was identified.

**Electronic supplementary material:**

The online version of this article (10.1007/s00125-019-4839-8) contains peer-reviewed but unedited supplementary material, which is available to authorised users.

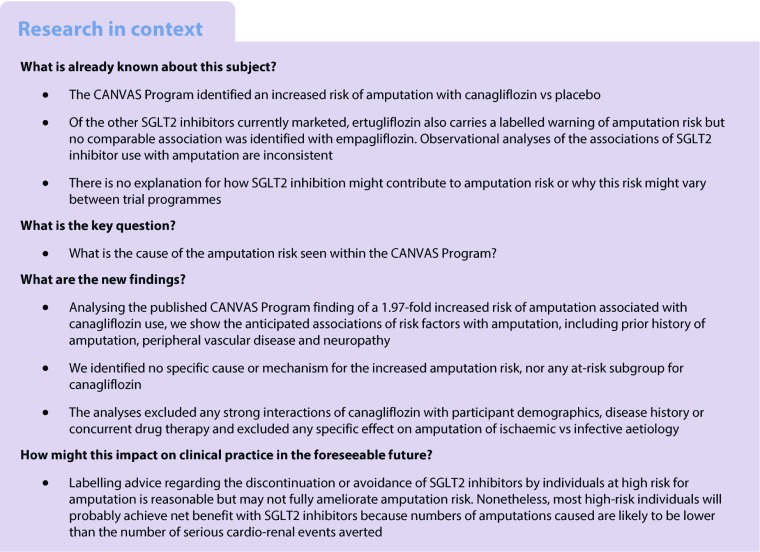



## Introduction

Canagliflozin is a sodium–glucose cotransporter 2 (SGLT2) inhibitor approved for the treatment of type 2 diabetes mellitus [1]. The Canagliflozin cardioVascular Assessment Study (CANVAS) Program [2] integrated data from two directly comparable, double-blind, randomised, placebo-controlled trials (CANVAS and CANVAS-Renal [CANVAS-R]; ClinicalTrials.gov registration no. NCT01032629, NCT01989754) [3, 4] involving participants with type 2 diabetes and a history or high risk of cardiovascular disease, conducted at 667 sites across 30 countries.

CANVAS was commenced in 2009 to demonstrate the cardiovascular safety of canagliflozin prior to registration, and CANVAS-R was commenced in 2014 as a post-registration commitment to regulatory agencies. The trials were designed to complete simultaneously when a prespecified minimum number of cardiovascular events (688) and a minimum follow-up time (78 weeks) were achieved; this occurred in late 2016 [5]. The CANVAS Program showed a beneficial effect of canagliflozin treatment on the primary outcome of major adverse cardiovascular events (HR 0.86 [95% CI 0.75, 0.97], *p* < 0.001 for non-inferiority, *p* < 0.02 for superiority) [2].

An emergent finding of the CANVAS Program was an increased risk of lower extremity major and minor amputation in participants treated with canagliflozin compared with placebo (HR 1.97 [95% CI 1.41, 2.75]) [2], where major atraumatic lower extremity amputations were defined as being at or above the ankle and minor as being atraumatic lower extremity amputations below the ankle. This safety signal was flagged to the Steering Committee in the final year of the trial by the Independent Data Monitoring Committee on 18 February 2016. Global health authorities, including those in Europe and the USA, were informed and responded with updates to the product information [6]. No prior clinical or preclinical study had raised any concern regarding limb perfusion or amputation, and the known tissue expression and actions of the SGLT isoforms provide no indication of the likely mechanism. Currently, of the seven marketed SGLT2 inhibitors, one other (ertugliflozin) also carries a labelled warning about amputation risk [7]. The objectives of this paper were to explore in more detail the effects of canagliflozin on amputation risk and to estimate likely risks and benefits for different participant subgroups.

## Methods

### Ethics

CANVAS and CANVAS-R were approved by the ethics committees at each site and all participants provided written informed consent. All procedures followed were in accordance with the Helsinki Declaration of 1964, as revised in 2013.

### Participants

Participants were men and women with type 2 diabetes mellitus and HbA_1c_ ≥53 mmol/mol (7.0%) and ≤91 mmol/mol (10.5%). They were ≥30 years of age with a history of symptomatic atherosclerotic cardiovascular disease (which included prior amputation) or ≥50 years of age with two or more of the following risk factors for cardiovascular disease: duration of diabetes ≥10 years, systolic BP >140 mmHg while on one or more antihypertensive agent, current smoker, microalbuminuria or macroalbuminuria or HDL-cholesterol <1 mmol/l [2]. The presence of amputation or peripheral vascular disease history at baseline was based upon physician reporting with no requirement for specific clinical evaluation or imaging.

### Randomised treatment

All potential participants completed a 2 week, single-blind, placebo run-in period with subsequent randomisation of eligible participants done centrally using an interactive web response system and a computer-generated randomisation schedule. Participants in CANVAS were randomly assigned in a 1:1:1 ratio to receive canagliflozin 300 mg, canagliflozin 100 mg or matching placebo, and participants in CANVAS-R were randomly assigned in a 1:1 ratio to receive canagliflozin or matching placebo (initially 100 mg with optional uptitration to 300 mg).

### Follow-up and data collection

Face-to-face follow-up was scheduled for three visits in the first year and at 6 month intervals thereafter, with alternating telephone follow-up between face-to-face assessments. Every follow-up included inquiry about serious adverse events. Amputation events were initially recorded routinely by the investigators using electronic case record forms for the capture of adverse events and/or diagnostic and therapeutic procedures. In July 2016, following notification of the amputation safety signal, a dedicated case report form was created to ensure systematic and standardised data collection for amputation events. Investigators were directed to complete the amputation case report form for every new event, as well as retrospectively for every amputation event already recorded. Additionally, all CANVAS Program participant case records and the associated pharmacovigilance database were searched using the terms ‘ampu’, ‘remov’, ‘resection’, ‘necrosectomy’, ‘disarticulation’, ‘exarticulation’, ‘BKA’ and ‘AKA’ to identify any additional possible amputation events. Information collected included the date of the amputation and whether the amputation was traumatic or atraumatic, as well as the location (right or left, toes, trans-metatarsal, ankle, below knee or above knee, other). Amputation events occurring at any time point prior to final follow-up, for all randomised participants regardless of whether the participant remained on the study drug, were captured [2]. A secondary post hoc assessment of the proximate aetiologies underlying each amputation was made by a specialist in peripheral vascular disease (MN), using source documentation collected at the time of the event from the participating sites, to identify the presence of infection, chronic ischaemia or acute ischaemia.

### Outcomes

In these secondary analyses, the main outcome was all atraumatic lower extremity amputations, which were reported as all major atraumatic lower extremity amputations (at or above ankle) and all minor atraumatic lower extremity amputations (below ankle). Benefits were reported for the composite outcome of major adverse cardiovascular events comprising cardiovascular death, non-fatal stroke and non-fatal myocardial infarction.

### Statistical methodology

The impact of canagliflozin on amputation risk was assessed among the dosed participants using a Cox proportional hazard model with treatment as the possible explanatory variable and adjustment for trial (CANVAS or CANVAS-R). The treatment effect was expressed as the HR and 95% CI, with a test of proportional hazards used to examine the evolution of the treatment effect over time. Comparability of the effects of canagliflozin on amputation was examined for participant subgroups by fitting treatment interaction terms to the proportional hazards models. Analyses were performed on the integrated CANVAS Program dataset except for the investigation of dose effects, which were done in the CANVAS dataset alone (without CANVAS-R). Baseline participant characteristics associated with amputation risk were assessed using proportional hazards models in the CANVAS Program dataset. First, univariate associations were determined for candidate risk factors for amputation independent of treatment assignment and then those risks with significant univariate associations were included in a single multivariate model that also included randomised treatment. Where a single risk was assessed using multiple measures (e.g. glucose and HbA_1c_), the risk with the greatest HR was carried forward for inclusion in the multivariate modelling. Absolute effects on all amputation, major amputation and major adverse cardiovascular events were modelled to estimate effects for 1000 participants treated for 5 years. This was done overall and for participant subsets defined by excluding participants with one or more of the risks for amputation that were significant in the multivariate modelling. The absolute effects on amputation and major adverse cardiovascular events were derived using the summary estimates of RRs derived from the CANVAS Program for each outcome applied to the absolute event rates specific to each subset of participants. All analyses were performed using SAS version 9.2 (SAS Institute, Cary, NC, USA), SAS Enterprise Guide version 7.1 (SAS Institute) and STATA version 13.1 (StataCorp, College Station, TX, USA).

## Results

The CANVAS Program randomised 10,142 participants (CANVAS [*n* = 4330] and CANVAS-R [*n* = 5812]) [3], of which 10,134 received at least one dose of randomised treatment. The mean follow-up of participants was 188 weeks (296 weeks in CANVAS and 108 weeks in CANVAS-R) [2]. Overall, the mean age of participants was 63.3 years, 35.8% were women, mean duration of diabetes was 13.5 years and 65.6% had a history of cardiovascular disease at baseline. Two hundred and thirty-eight (2%) participants had a history of major or minor amputation at baseline and 187 (1.8%) had one or more major or minor amputation events during follow-up. In total, 290 amputation events were recorded among these 187 participants during follow-up.

### Associations of baseline participant characteristics with amputation risk

At trial commencement, participants who had major or minor amputation during follow-up differed from participants who did not go on to have amputation during the trial (Table 1). Univariate modelling identified more than 20 baseline characteristics that were associated with amputation risk, eight of which (male sex, non-Asian ethnicity, prior amputation, peripheral vascular disease, neuropathy, albuminuria, higher HbA_1c_ and random allocation to canagliflozin) remained significant in multivariate modelling (Table 2).

**Table 1:** Baseline characteristics of participants with and without major or minor amputation during follow-up^a^

Characteristic	Participants with amputation			Participants without amputation			*p* value (total with amputation vs total without amputation)^b^
	Canagliflozin (*n* = 140)	Placebo (*n* = 47)	Total (*n* = 187)	Canagliflozin (*n* = 5650)	Placebo (*n* = 4297)	Total (*n* = 9947)	
Age, years, mean (SD)	62.3 (7.1)	60.9 (6.3)	62.0 (6.9)	63.2 (8.3)	63.5 (8.2)	63.3 (8.3)	0.025
Female sex, *n* (%)	27 (19.3)	5 (10.6)	32 (17.1)	2007 (35.5)	1592 (37.0)	3599 (36.2)	<0.001
Race, *n* (%)							0.008
White	120 (85.7)	44 (93.6)	164 (87.7)	4385 (77.6)	3389 (78.9)	7774 (78.2)	
Asian	8 (5.7)	2 (4.3)	10 (5.3)	769 (13.6)	505 (11.7)	1274 (12.8)	
Black or African-American	2 (1.4)	1 (2.1)	3 (1.6)	173 (3.1)	159 (3.7)	332 (3.3)	
Other^c^	10 (7.1)	0 (0.0)	10 (5.3)	323 (5.7)	244 (5.7)	567 (5.7)	
Current smoker, *n* (%)	22 (15.7)	14 (29.8)	36 (19.3)	996 (17.6)	770 (17.9)	1766 (17.8)	0.597
History of hypertension, *n* (%)	123 (87.9)	42 (89.4)	165 (88.2)	5060 (89.6)	3893 (90.6)	8953 (90.0)	0.424
Duration of diabetes, years, mean (SD)	16.8 (8.6)	14.8 (8.4)	16.3 (8.6)	13.4 (7.7)	13.7 (7.8)	13.5 (7.7)	<0.001
Microvascular disease history, *n* (%)							
Nephropathy	40 (28.6)	16 (34.0)	56 (29.9)	953 (16.9)	763 (17.8)	1716 (17.3)	<0.001
Retinopathy	50 (35.7)	19 (40.4)	69 (36.9)	1152 (20.4)	906 (21.1)	2058 (20.7)	<0.001
Neuropathy	84 (60.0)	27 (57.4)	111 (59.4)	1703 (30.1)	1295 (30.1)	2998 (30.1)	<0.001
Atherosclerotic disease, *n* (%)^d^							
Coronary	83 (59.3)	28 (59.6)	111 (59.4)	3148 (55.7)	2458 (57.2)	5606 (56.4)	0.413
Cerebrovascular	35 (25.0)	10 (21.3)	45 (24.1)	1076 (19.0)	835 (19.4)	1911 (19.2)	0.111
Peripheral	81 (57.9)	32 (68.1)	113 (60.4)	1094 (19.4)	904 (21.0)	1998 (20.1)	<0.001
Any	129 (92.1)	43 (91.5)	172 (92.0)	3994 (70.7)	3152 (73.4)	7146 (71.8)	<0.001
History of cardiovascular disease, *n* (%)^e^	116 (82.9)	38 (80.9)	154 (82.4)	3636 (64.4)	2861 (66.6)	6497 (65.3)	<0.001
History of atrial fibrillation, *n* (%)	12 (8.6)	6 (12.8)	18 (9.6)	339 (6.0)	256 (6.0)	595 (6.0)	0.038
History of heart failure, *n* (%)	27 (19.3)	8 (17.0)	35 (18.7)	774 (13.7)	650 (15.1)	1424 (14.3)	0.093
History of amputation, *n* (%)	38 (27.1)	13 (27.7)	51 (27.3)	98 (1.7)	88 (2.0)	186 (1.9)	<0.001
BMI, kg/m^2^, mean (SD)	32.5 (5.9)	33.3 (6.9)	32.7 (6.1)	31.9 (5.9)	32.0 (5.9)	31.9 (5.9)	0.0765
Systolic BP, mmHg, mean (SD)	138.5 (16.4)	135.0 (15.7)	137.6 (16.3)	136.4 (15.8)	136.9 (15.8)	136.6 (15.8)	0.3947
Diastolic BP, mmHg, mean (SD)	77.3 (9.4)	78.0 (10.1)	77.5 (9.6)	77.6 (9.6)	77.8 (9.7)	77.7 (9.7)	0.7711
HbA_1c_, mmol/mol, mean (SD)	69 (9.8)	68 (10.9)	69 (9.8)	66 (9.8)	66 (9.8)	66 (9.8)	<0.001
HbA_1c_, %, mean (SD)	8.5 (0.9)	8.4 (1.0)	8.5 (0.9)	8.2 (0.9)	8.2 (0.9)	8.2 (0.9)	<0.001
LDL-cholesterol, mmol/l, mean (SD)	2.3 (1.0)	2.5 (0.9)	2.4 (1.0)	2.3 (0.9)	2.3 (0.9)	2.3 (0.9)	0.3481
LDL/HDL-cholesterol ratio, mean (SD)	2.1 (1.0)	2.3 (0.8)	2.1 (0.9)	2.0 (0.9)	2.0 (0.9)	2.0 (0.9)	0.1537
eGFR, ml min^−1^ [1.73 m]^−2^, mean (SD)^f^	72.4 (18.2)	73.7 (23.5)	72.7 (19.7)	76.8 (20.3)	76.2 (20.8)	76.5 (20.5)	0.0121
Micro- or macroalbuminuria, *n* (%)^g^	69 (49.6)	26 (56.5)	95 (51.4)	1656 (29.6)	1272 (30.0)	2928 (29.7)	<0.001
Concomitant drug therapies, *n* (%)							
Insulin	96 (68.6)	35 (74.5)	131 (70.1)	2793 (49.4)	2169 (50.5)	4962 (49.9)	<0.001
Metformin	92 (65.7)	37 (78.7)	129 (69.0)	4351 (77.0)	3340 (77.7)	7691 (77.3)	0.0071
Sulfonylurea	51 (36.4)	18 (38.3)	69 (36.9)	2475 (43.8)	1815 (42.2)	4290 (43.1)	0.0882
GLP-1 receptor agonist	8 (5.7)	2 (4.3)	10 (5.3)	214 (3.8)	183 (4.3)	397 (4.0)	0.3493
DPP-4 inhibitor	12 (8.6)	5 (10.6)	17 (9.1)	685 (12.1)	559 (13.0)	1244 (12.5)	0.1610
Loop diuretic	33 (23.6)	8 (17.0)	41 (21.9)	683 (12.1)	584 (13.6)	1267 (12.7)	0.0002
Non-loop diuretic	53 (37.9)	17 (36.2)	70 (37.4)	2030 (35.9)	1546 (36.0)	3576 (36.0)	0.6756
Calcium antagonist	52 (37.1)	17 (36.2)	69 (36.9)	1878 (33.2)	1496 (34.8)	3374 (33.9)	0.3942
RAAS inhibitor	112 (80.0)	36 (76.6)	148 (79.1)	4530 (80.2)	3435 (79.9)	7965 (80.1)	0.7525
β-Blocker	79 (56.4)	30 (63.8)	109 (58.3)	2959 (52.4)	2352 (54.7)	5311 (53.4)	0.1836
Statin	102 (72.9)	35 (74.5)	137 (73.3)	4224 (74.8)	3235 (75.3)	7459 (75.0)	0.5895
Aspirin	67 (47.9)	20 (42.6)	87 (46.5)	1884 (33.3)	978 (22.8)	2862 (28.8)	<0.001
Other antithrombotic	41 (29.3)	24 (51.1)	65 (34.8)	2240 (39.6)	2213 (51.5)	4453 (44.8)	0.006

^a^One participant was randomised at two different sites and only the first randomisation is included in the intention-to-treat analysis set

^b^Analysed with a Wilcoxon two-sample test

^c^Includes American Indian or Alaska Native, Native Hawaiian or other Pacific Islander, multiple, other and unknown

^d^Some participants had >1 type of atherosclerotic disease

^e^As defined in the protocol

^f^Values for eGFR categories calculated based on *N* of 5794 for canagliflozin, 4346 for placebo and 10,140 for the total population

^g^Values for albuminuria categories calculated based on *N* of 5740 for canagliflozin, 4293 for placebo and 10,033 for the total population

DPP-4, dipeptidyl pepdidase-4; RAAS, renin–angiotensin–aldosterone system

**Table 2:** Association of baseline participant characteristics with risk of major or minor amputation in univariate and multivariate models

Characteristic	Univariate	Multivariate
Demographics		
Male sex	2.63 (1.80, 3.85)	2.26 (1.53, 3.35)
Age (year older)	0.98 (0.97, 1.00)	
Current smoker	1.11 (0.77, 1.59)	
Race		
White vs non-white	2.18 (1.41, 3.38)	
Asian vs non-Asian	0.32 (0.17, 0.61)	0.44 (0.23, 0.85)
Black vs non-Black	0.59 (0.19, 1.86)	
Region		
North America vs others	1.07 (0.77, 1.48)	
Central/South America vs others	1.64 (1.03, 2.62)	
Europe vs others	1.13 (0.84, 1.52)	
Rest of world vs others	0.68 (0.49, 0.94)	
Prior amputation (Yes/No)	21.31 (15.40, 29.49)	16.27 (10.65, 24.63)
Peripheral vascular disease (Yes/No)^a^	2.51 (1.85, 3.41)	2.77 (1.93, 3.96)
Cardiovascular disease (Yes/No)	2.85 (1.95, 4.16)	
Microvascular disease history		
Neuropathy (Yes/No)	3.38 (2.52, 4.52)	1.86 (1.35, 2.56)
Nephropathy (Yes/No)	2.18 (1.60, 2.99)	
Retinopathy (Yes/No)	2.27 (1.69, 3.06)	
Any albuminuria (Yes/No)	2.65 (1.99, 3.54)	1.63 (1.20, 2.22)
Hypertension (Yes/No)	0.91 (0.58, 1.42)	
Heart failure (Yes/No)	1.52 (1.05, 2.19)	
Duration of diabetes (year greater)	1.04 (1.03, 1.06)	
Concomitant medications (Yes/No)		
Insulin use	2.37 (1.73, 3.24)	
Sulfonylurea	0.72 (0.53, 0.97)	
Metformin	0.68 (0.50, 0.93)	
GLP-1 receptor agonist	1.59 (0.84, 3.03)	
DPP-4 inhibitor	0.83 (0.50, 1.37)	
Loop diuretic	1.26 (0.95, 1.68)	
Non-loop diuretic	1.04 (0.77, 1.40)	
Statin	0.94 (0.68, 1.30)	
Aspirin	1.34 (0.89, 2.03)	
Other antithrombotic	1.70 (1.04, 2.80)	
RAAS inhibitor	0.93 (0.66, 1.33)	
β-Blocker	1.28 (0.96, 1.71)	
Calcium channel blocker	1.17 (0.87, 1.57)	
Laboratory and clinical variables		
HbA_1c_ (10.9 mmol/mol [1%] greater)	1.37 (1.18, 1.58)	
HbA_1c_ (≥64 mmol/mol [8%] vs <64 mmol/mol [8%])	2.12 (1.54, 2.93)	1.99 (1.43, 2.76)
Haemoglobin (1 g/l greater)	1.00 (0.99, 1.01)	
eGFR (1 ml/min greater)	0.99 (0.98, 1.00)	
Systolic BP (1 mmHg greater)	1.00 (1.00, 1.01)	
Haematocrit (1% greater)	1.00 (0.97, 1.04)	
BMI (1 kg/m^2^ greater)	1.02 (1.00, 1.04)	
LDL-cholesterol (0.026 mmol/l greater)	1.08 (0.93, 1.26)	
HDL-cholesterol (0.026 mmol/l greater)	0.74 (0.45, 1.19)	
Triacylglycerols (0.011 mmol/l greater)	1.07 (1.00, 1.15)	
Canagliflozin treatment	1.97 (1.41, 2.75)	1.82 (1.29, 2.56)

Data are shown as HR (95% CI)

The multivariate model included all characteristics with significant univariate associations. Only characteristics significant in the multivariate model are listed

^a^Excluding history of amputation

DPP-4, dipeptidyl peptidase-4; RAAS, renin–angiotensin–aldosterone system

### Proximate aetiology of amputation

Among the 187 participants who underwent a post-randomisation amputation, infection was identified as a proximate aetiology in most events: 136/140 (97%) on canagliflozin and 47/47 (100%) on placebo. About two-thirds also had chronic ischaemia: 82/140 (59%) on canagliflozin and 31/47 (66%) placebo. Only 2/140 (1.4%) canagliflozin-treated cases had an acute ischaemic aetiology recorded. Of the 187 participants experiencing amputation, 140 (75%) were using randomised treatment at the time of amputation, 19 (10%) had amputations within 30 days after discontinuation of randomised treatment and 28 (15%) had amputations more than 30 days after discontinuation of randomised treatment.

### Effects of canagliflozin on the risk of amputation

**Fig. 1:**
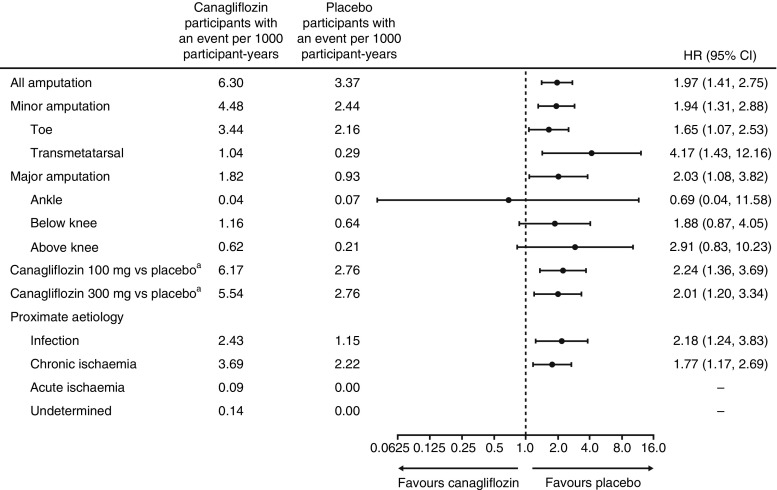
Risk of amputation with canagliflozin compared with placebo, overall, based on the highest level of amputation, according to each dose of canagliflozin or presumed aetiology. ^a^Based on CANVAS data alone

**Fig. 2.**
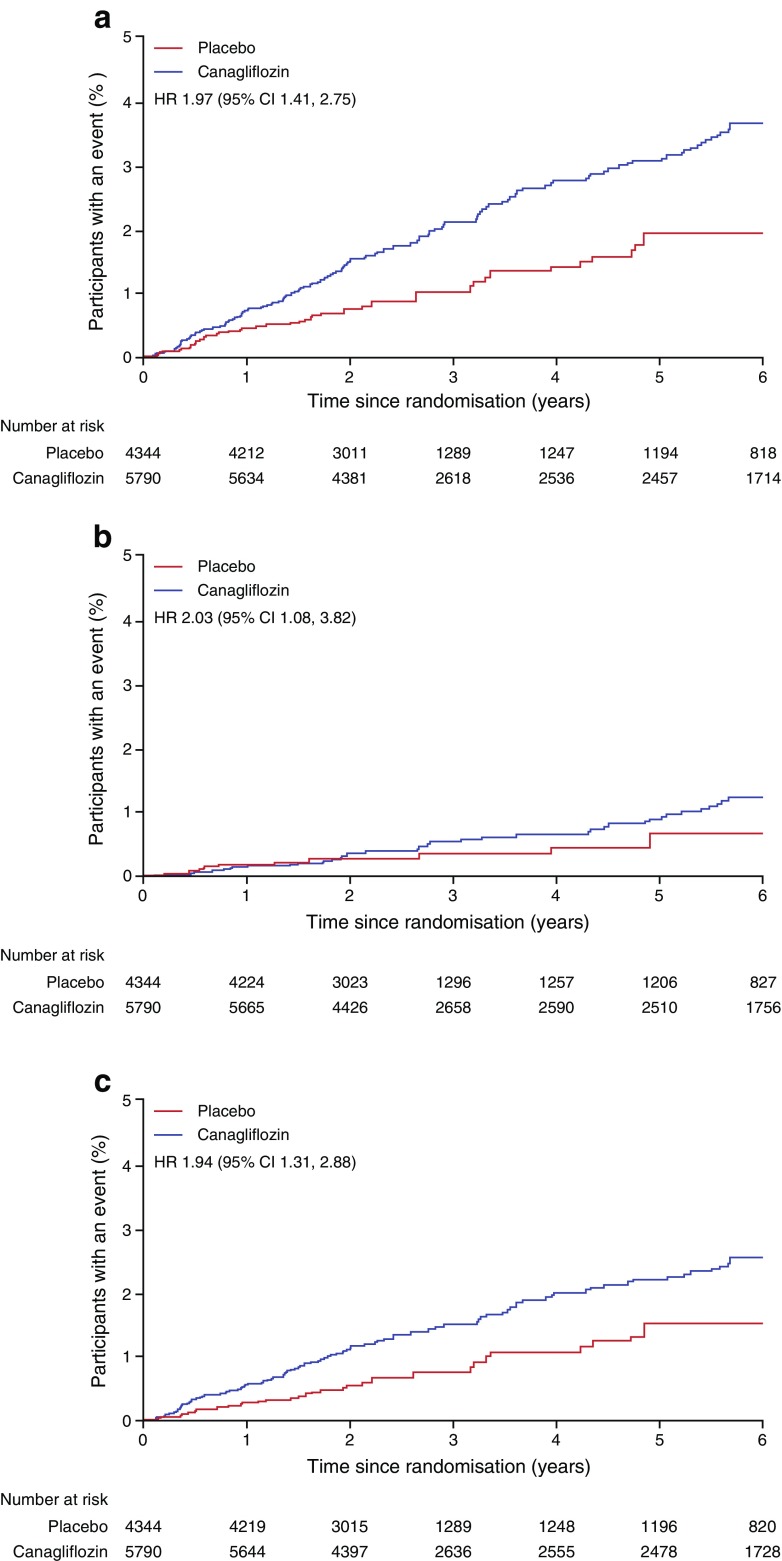
Effects of canagliflozin compared with placebo on the risk of any amputation (**a**), major amputation (**b**) and minor amputation (**c**)

**Fig. 3.**
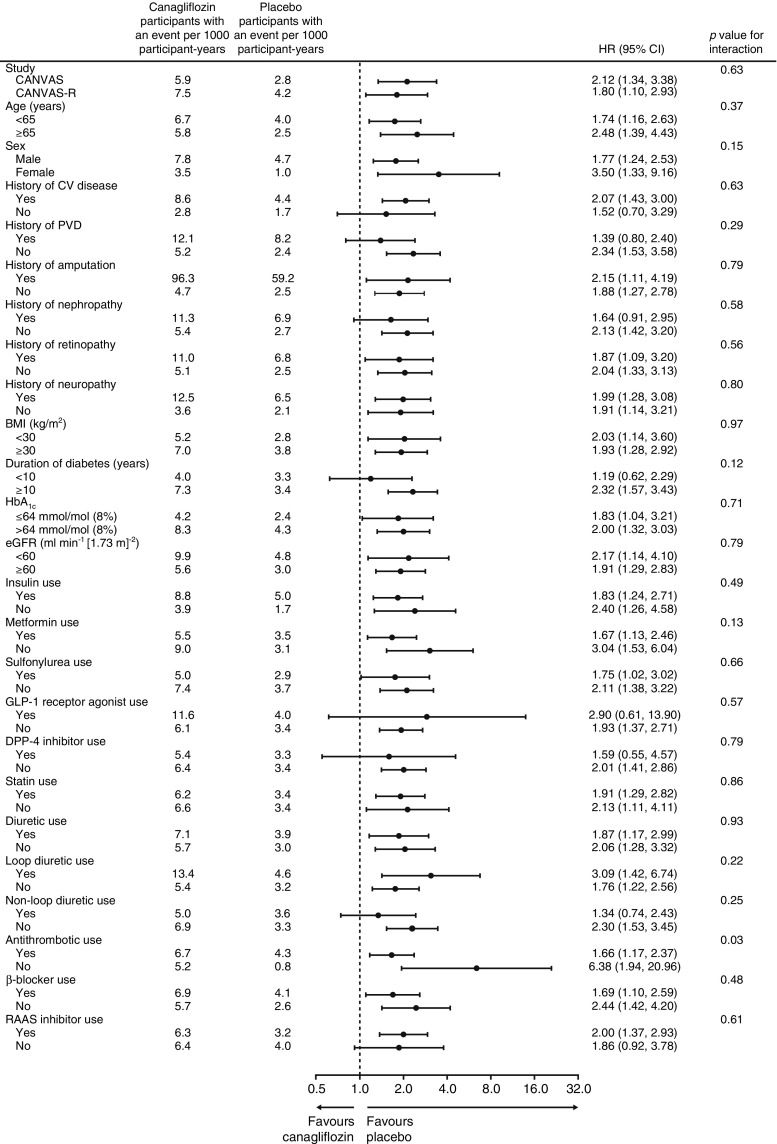
Risk of amputation with canagliflozin compared with placebo in participant subgroups. CV, cardiovascular; DPP-4, dipeptidyl peptidase-4; PVD, peripheral vascular disease; RAAS, renin–angiotensin–aldosterone system

Major or minor amputation events occurred in 47 of 4344 participants treated with placebo and 140 of 5790 participants treated with canagliflozin. The corresponding rates of major or minor amputation events were 3.37 and 6.30 events per 1000 participant-years and the HR was 1.97 (95% CI 1.41, 2.75) (Figs 1 and 2). The difference in the risk of amputation events between randomised groups evolved progressively throughout the study (test for proportional hazards *p* = 0.88), with similar patterns of accrual observed for total, major and minor amputations (Fig. 2). There was no evidence that the effects of canagliflozin on amputation risk varied according to any baseline participant characteristic (all *p* homogeneity >0.123). A possible exception was use of antithrombotic therapy, where the effects of canagliflozin compared with placebo were conventionally significantly greater among those not using such treatment (*p* = 0.0268). However, considering that small numbers were involved and that 25 interactions were tested, it is highly likely that one or more would meet the criterion for significance based on chance alone (Fig. 3).

Of the amputations recorded, 71% were minor and 29% major. There were no differences in proportional effects on the risks of minor vs major amputation (Fig. 1). The 100 mg and 300 mg doses of canagliflozin had similar effects on amputation risk compared with placebo in the CANVAS participants who were randomised between doses. The majority of participants who experienced amputation had a single event recorded (*n* = 123), and proportional effects of canagliflozin vs placebo on amputation risk were comparable with those experiencing more than one amputation event (*n* = 64). There were multiple amputations at varying locations on the same limb in 22% of participants assigned canagliflozin and 19% of participants assigned placebo and amputations at varying locations in both limbs for 13% of participants assigned canagliflozin and 17% of participants assigned placebo.

### Risk of amputation and protection against major adverse cardiovascular events in participant groups without baseline risks for amputation

Among the overall CANVAS population, there were four (95% CI 1, 8) more major amputations among every 1000 participants treated for 5 years with canagliflozin compared with placebo and 15 (95% CI 8, 22) more amputations in total (major or minor) (Table 3). There were 23 fewer major adverse cardiovascular events among every 1000 participants treated for 5 years (95% CI 4, 42). The estimated numbers of excess amputations with canagliflozin compared with placebo were lower for major and all amputations among most participant subsets where there were baseline risks for amputation identified from the multivariate modelling (male sex, non-Asian ethnicity, prior amputation peripheral vascular disease, neuropathy, albuminuria and higher HbA_1c_) (Table 3). The estimated absolute reductions in major adverse cardiovascular events were also lower in those taking canagliflozin compared with placebo in most of these participant subsets. The same pattern of greater and lesser numbers of events with canagliflozin compared with placebo was also apparent for participant subsets derived by exclusions based upon combinations of the identified baseline risks for amputation (Table 3).

**Table 3:** Numbers of major or minor amputations and major adverse cardiovascular events for every 1000 participants treated for 5 years, overall, and after exclusion of participant subsets with risks for amputation

Risk factor	Participants excluded *n* (%)	Participants with an amputation *n* events per 1000 participant-years		Amputations among 1000 participants treated for 5 years *n* (95% CI)		Major adverse cardiovascular events prevented among 1000 participants treated for 5 years *n* (95% CI)
		Canagliflozin	Placebo	All	Major	
All participants	0 (0)	6.3	3.4	15 (8, 22)	4 (1, 8)	23 (4, 41)
Excluding those with baseline risks identified as independent in multivariate modelling						
Amputation	237 (2)	4.7	2.5	11 (5, 17)	5 (2, 8)	21 (3, 40)
Macroalbuminuria	865 (9)	5.5	2.9	13 (6, 20)	4 (1, 8)	17 (–2, 35)
Male sex	6503 (64)	3.5	1.0	13 (5, 20)	4 (–1, 9)	17 (–11, 45)
Non-Asian ethnicity	8850 (87)	2.3	1.0	6 (–4, 17)	4 (–1, 9)	–2 (–39, 36)
PVD^a^	1874 (18)	5.2	2.4	14 (7, 21)	3 (–1, 7)	19 (0, 39)
Neuropathy	3109 (31)	3.6	2.0	8 (1, 14)	3 (–1, 6)	28 (7, 49)
HbA_1c_ ≥64 mmol/mol (8%)	5729 (57)	3.6	2.5	5 (–3, 14)	3 (–2, 7)	11 (–14, 37)
Excluding those with baseline risk combinations						
Amputation or macroalbuminuria	937 (9)	4.2	2.3	10 (4, 16)	5 (2, 8)	15 (–4, 33)
PVD or macroalbuminuria	2467 (24)	4.5	2.0	13 (6, 19)	3 (–1, 6)	13 (–7, 33)
Amputation or PVD	2111 (21)	3.2	1.3	10 (4, 15)	3 (0, 6)	17 (–2, 37)
Neuropathy or macroalbuminuria	3588 (35)	3.1	1.4	8 (2, 14)	3 (0, 7)	12 (0, 24)
Amputation or neuropathy	3211 (32)	2.7	1.6	6 (0, 12)	3 (0, 6)	26 (5, 47)
PVD or neuropathy	4037 (40)	3.0	1.2	9 (3, 15)	2 (–1, 4)	23 (1, 44)
Amputation, PVD or macroalbuminuria	2644 (26)	2.9	1.2	9 (3, 14)	3 (0, 6)	11 (–8, 310)
Amputation, neuropathy or macroalbuminuria	3664 (36)	2.3	1.3	5 (0, 10)	3 (0, 6)	19 (–2, 40)
PVD, neuropathy or macroalbuminuria	4438 (44)	2.5	0.7	9 (4, 14)	2 (0, 5)	18 (–4, 39)
Amputation, PVD or neuropathy	4139 (41)	2.0	0.6	7 (2, 12)	2 (0, 4)	21 (–1, 43)
Amputation, PVD, neuropathy or macroalbuminuria	4512 (45)	1.7	0.6	5 (1, 10)	2 (–1, 4)	16 (–6, 38)

Numbers of events caused and prevented were estimated using the summary CANVAS Program estimates of relative risks for each outcome, but the absolute event rates are those specific to each participant subgroup

^a^Excluding those with amputation history

PVD, peripheral vascular disease

## Discussion

We have previously reported the main outcomes of the CANVAS Program, which identified protective effects of canagliflozin against cardiovascular and renal outcomes but also demonstrated an increased risk of major and minor amputation [2]. This paper reports on our investigation of possible explanatory aetiological factors related to the excess amputation rate seen for both minor and major amputations. Overall, the absolute risk of major amputation was low and was offset by improvements in cardiovascular outcomes. Minor amputation, defined as surgery below the ankle, was more than twice as common relative to major amputation (at or above the ankle) but was also offset by the cardiovascular benefits. Neither a proximate aetiology nor an explicit risk category for the excess risk of amputation with canagliflozin compared with placebo could be defined. The excess risk was found across all examined categories.

Identified independent predictors of amputation within the CANVAS Program were a prior history of amputation, male sex, race, history of peripheral vascular disease, history of neuropathy, albuminuria and higher pre-randomisation levels of HbA_1c_ in addition to treatment with canagliflozin. Of these, the highest risk was prior amputation, which we show carries an estimated 21.31-fold risk using univariate modelling and an estimated 16.27-fold risk using multivariate analyses. This compares with canagliflozin use yielding a 1.97-fold and 1.82-fold risk, respectively. There were no differences in the proportional risks of amputation associated with canagliflozin across participant subgroups defined by the presence or absence of these or most other baseline risks. The proportional risks of amputation associated with canagliflozin were also consistent across different subsets of amputation defined on the basis of site, severity and proximate aetiology. Ultimately, it was not possible to specify characteristics that could precisely identify the subset of CANVAS participants who had amputation events, and it was not possible to further specify which individuals would achieve the optimal balance of benefit and risk with canagliflozin treatment. Existing recommendations pertaining to the careful monitoring of individuals with a higher risk for amputation events and provision of counselling about the importance of routine preventative foot care stand, as does advice regarding the possible discontinuation of canagliflozin among those who develop diabetic foot ulcers, infection, osteomyelitis or gangrene. The current data add weight to these recommendations.

There are few additional amputation events recorded in other phase 3 and 4 randomised trials of canagliflozin vs placebo or active comparators (*n* = 10/8114, RR 0.23, 95% CI 0.06, 0.89), reflecting both the lower cardiovascular risk of those participants and a shorter average duration of follow-up [8]. Empagliflozin displayed similar rates of amputation when compared with placebo (6.5 per 1000 participant-years for both treatment groups) in EMPA-REG OUTCOME [9], with an overall RR for amputation with empagliflozin vs placebo of 1.02 (95% CI 0.72, 1.45) [10]; comparable rates in the CANVAS Program were 6.30 (active) and 3.37 (placebo) per 1000 participant-years. Recent data for ertugliflozin [7] suggest an imbalance in atraumatic lower limb amputation, with rates per 1000 participant-years in the 15 mg, 5 mg and comparator groups of 4.4, 1.6 and 0.6 across the development programme (12 events) and 5.0, 6.8 and 4.3 in the separate and ongoing cardiovascular outcome trial (61 events). Additional data for these and other SGLT2 inhibitors under investigation in large-scale trials remain undisclosed at this time. The ongoing Canagliflozin and Renal Events in Diabetes with Established Nephropathy Clinical Evaluation (CREDENCE) trial of canagliflozin among individuals with nephropathy [11] will provide valuable additional insight about the effects of canagliflozin on amputation.

In an observational analysis of the Truven United States Commercial database (*n* = 63,845 matched pairs), new users of canagliflozin were not observed to have increased risks of amputation compared with new users of non-SGLT2 glucose-lowering therapies (HR 0.98 [95% CI 0.68, 1.41]) [8]. The same was true in the OBSERVE-4D analysis of 142,800 new users of canagliflozin compared with other agents [12]. By contrast, data from the Department of Defense database [13] showed that individuals with type 2 diabetes and established cardiovascular disease treated with SGLT2 inhibitors (mostly canagliflozin) compared with other glucose-lowering therapies (*n* = 12,629 matched pairs) had an increased risk of amputation (HR 1.99 [95% CI 1.12, 3.51]) directly comparable in magnitude with that observed in the CANVAS Program. A disproportionality analysis of the WHO global database of individual case safety reports (VigiBase) based upon 79 amputation events identified increased proportional reporting ratios for SGLT2 inhibitors that were significant for canagliflozin, empagliflozin and dapagliflozin [14, 15]. A registry database from Sweden and Denmark, using a propensity score matched cohort of 17,213 new users of SGLT2 inhibitors (dapagliflozin 61%; empagliflozin 38%; canagliflozin 1%) and 17,213 new users of an active glucagon-like peptide-1 (GLP-1) receptor agonist demonstrated an HR for amputation of 2.32 (95% CI 1.37, 3.91) for SGLT2 inhibitors [16].

Based primarily upon the CANVAS Program findings, the US Food and Drug Administration [17] and the European Medicines Agency [18] updated the product information of canagliflozin to reflect amputation risk. Warnings related to amputation appear in the product information for canagliflozin and ertugliflozin but not empagliflozin and dapagliflozin. The large-scale EMPA-REG OUTCOME study [19] did not initially report upon amputation risk, but retrospective analyses of the trial and the broader empagliflozin database identified no relative differences in amputation rates between individuals on active therapy and those on placebo [20]. The canagliflozin and ertugliflozin reports benefitted from data collected as part of active trial programmes, rather than retrospective investigations of the databases; whether this or unknown differences in the pharmacological actions of the compounds explains the different findings from empagliflozin is uncertain. Additional prospectively collected data for amputation will become available from ongoing large trials of these and other SGLT2 inhibitors over the next few years, and will be key to understanding whether the amputation risk is specific to particular drugs or is a class effect and whether the risk can be ameliorated through specific active management strategies.

The present report benefits from the rigorous design, conduct and analysis of the CANVAS Program, the rigorous search of the database for all possible amputation events and the careful masked adjudication of identified events. However, there was limited documentation of peripheral artery disease at baseline and incomplete recording of detailed description of the acute and chronic factors leading to each amputation. The few amputation events recorded limited the capacity to detect effects in participant subgroups and interpreting multiple tests makes interpretation of borderline significant findings difficult (e.g. the interaction of canagliflozin treatment and amputation risk with baseline antithrombotic use). Likewise, the small numbers of events weaken the conclusions about the subsets of amputation events. The absence of a clear mechanism by which canagliflozin or any other SGLT2 inhibitor might contribute to amputation also adds significant complexity to the interpretation of the findings.

In conclusion, the CANVAS Program demonstrated that canagliflozin increased the risk of amputation (mainly minor) in this study population. Anticipated risk factors for amputation, such as prior history of amputation, peripheral vascular disease and neuropathy, were identified (see Table 3) but no specific aetiological mechanism or at-risk subgroup for canagliflozin was identified. Despite that increased risk, however, canagliflozin showed significant protective effects against major cardiovascular events and demonstrated HRs indicative of reduction of renal complications in type 2 diabetes.

## Electronic supplementary material

ESM Appendix (PDF 143 kb)

## Data Availability

Data from the CANVAS Program will be made available in the public domain via the Yale University Open Data Access Project (YODA; http://yoda.yale.edu/) once the product and relevant indication studied have been approved by regulators in Europe and the USA and the study has been completed for 18 months.

## Abbreviations

CANVAS: Canagliflozin cardioVascular Assessment Study
CANVAS-R: CANVAS-Renal
GLP-1: Glucagon-like peptide-1
SGLT2: Sodium–glucose cotransporter 2
