# Supplementary material for: Effects of canagliflozin on amputation risk in type 2 diabetes: the CANVAS Program
Source: Diabetologia. 2019 Mar 12;62(6):926–38. doi: 10.1007/s00125-019-4839-8 (PMC6509073; doi:10.1007/s00125-019-4839-8)
Supplement: Supplementary file 1 — (PDF 143 kb) [file 125_2019_4839_MOESM1_ESM.pdf]

Supplementary Appendix

CANVAS and CANVAS-R sites and investigators

CANVAS

Argentina: Pablo Arias, Maria Rosa Ulla, Andres Alvarisqueta, Laura Maffei, Jose Osvaldo Fretes, Silvia Gorban De Lapertosa, Virginia Visco, Georgina Sposetti, Javier Farias, Eduardo Francisco Farias, Maria Cecilia Cantero, Rodolfo Feldman, Maria Carolina Ridruejo, Pedro Calella, Cesar Zaidman; **Australia:** Stephen Stranks, Peak Man Mah, Alison Nankervis, Duncan Topliss, Georgia Soldatos, Richard Simpson, Murray Gerstman, David Colquhoun, Ferdinandus De Looze, Robert Moses, Michael Suranyi, Samantha Hocking, David Packham, Duncan Cooke, Karam Kostner; **Belgium:** Eric Weber, Chris Vercammen, Luc Van Gaal, Jozef Tits, Bart Keymeulen, Chantal Mathieu; **Canada:** Naresh Aggarwal, Dan Dattani, Francois Blouin, Richard Dumas, Sam Henein, Patrick Ma, Ali Najarali, Michael Omahony, Tracy Pella, Wilson Rodger, Daniel Shu, Vincent Woo, Brian Zidel, Lew Pliamm, Brian Ramjattan, Ronald Akhras, Jasmin Belle-Isle, Stuart Ross, Geza Molnar; **Colombia:** Juan Manual Arteaga, Ivonne Jarava; **Czech Republic:** Alena Andresova, Miloslava Komrskova, Cyril Mucha, Tomas Brychta, Dagmar Bartaskova, Romana Urbanova, Tomas Spousta, Jana Havelkova, Tomas Sedlacek, Milan Kvapil; **Estonia:** Ülle Jakovlev, Verner Fogel, Liina Viitas, Mai Soots, Maire Lubi, Marju Past, Jelena Krasnopejeva; **Germany:** Hasan Alawi, Klaus Busch, Felix Klemens Pröpper, Andrea Thron, Stephan Jacob, Andreas Pfützner, Ludger Rose, Thomas Segiet, Christine Kosch, Andrea Moelle; **Great Britain:** Melanie Davies, Hamish Courtney, Martin Gibson, Luigi Gnudi, Frances Game, John Wilding, Thozhukat Sathyapalan, Miles Fisher, Shenaz Ramtoola, Satyan Rajbhandari, Maurice Okane; **Hungary:** Eleonora Beke, Ferenc Poor, Karoly Nagy, Gyozo Kocsis, Tamas Oroszlan, Peter Faludi, Mihaly Gurzo; **India:** Sathyanarayana Srikanta, Mala Dharmalingam, Bala Murugan, Pramod Gandhi, Bipin Sethi, Sosale Aravind, Sharda Ardhanareeshwaran, Arpan Bhattacharyya, Ganapathi Bantwal, Vijay Viswanathan, Paramesh Shamanna, Banshi Saboo, Viswanathan Mohan, Reshma Parmaj, Kirti Kumar Modi, Sindhu Joshi, Sunil Jain, Sanjay Kalra, Arun Chankramath Somasekharan, Prabha Adhikari, Ajay Kumar, Harshada Kudalkar, Rajiv Passey, Mathew John, Sadasivarao Yalamanchi, Keyur Parikh, K.P. Rajesh, Rajesh Nair, Ajay Kumar, Sasi Kumar, Lily Rodrigues, Pawan Gangwal, Pankaj Agarwal, Sandeep Kumar Gupta, Abhay Amrutlal Mutha, Shailaja Dilip Kale, Ravindra Laxman Kulkarni, Sandip Chudasama, Kamal Sharma, Anoop Nambiar, Aniruddha Tangaonkar, Vaishali Deshmukh, Biswakesh Majumdar, Rajendran Veerappan, Deepak Namjoshi; **Israel:** Itamar Raz, Julio Weinstein, Ilana Harman Boehm, Victor Vishlitzky; **Luxembourg:** Frederic Dadoun; **Malaysia:** Rajesh P. Shah, Lai Seong Hooi, Alexander Tan, Wan Mohamad Wan Bebakar, Mafauzy Mohamed, Amir S. Khir, Norlela Sukor, Khalid Abdul Kadir; **Mexico:** Enrique Morales, Sergio Zuñiga, Melchor Alpizar, Cesar Calvo, Rolando Zamarripa, Juan Rosas, Armando Vargas; **The Netherlands:** Max Nieuwdorp, Vicdan Kose, Susanne Kentgens, Gloria Rojas, Wouter Van Kempen, Jacqueline Hoogendijk, Mazin Alhakim, Victor Gerdes, Marcel Hovens, Johan Berends, A. Woittiez, Cees Jan Smit, B. Dekkers, Wilco Spiering, Marcel K. Van Dijk-Okla, Ben P.M. Imholz, Ruud J.M. Van Leendert, Marije Ten Wolde, Peter J.H. Smak Gregoor; **New Zealand:** Russell Scott, Jeremy Krebs, John Baker, Joe Singh, Calum Young; **Norway:** Gisle Langslet, Hans Olav Hoivik, Torbjorn Kjaernli, Sigbjorn Elle, Eric Gjertsen, Knut Risberg, Andreas Tandberg, Leidulv Solnoer, Per Anton Sirnes; **Poland:** Tadeusz Derezinski, Malgorzata Arciszewska, Edward Franek, Ewa Szyprowska, Dariusz Sowinski, Robert Petryka, Beata Czakanska-Dec, Grazyna Pulka, Katarzyna Jusiak, Mariusz Dabrowski, Piotr Kubalski, Malgorzata Wojciechowska, Andrzej Madej, Danuta Pupek-Musialik; **Russia:** Natalia Blinova, Ludmila Kondratjeva, Anatoly Kuzin, Mikhail Boyarkin, Tatyana Gomova, Alexander Khokhlov, Sergey Vorobjev, Olga Mirolyubova, Svetlana Boldueva, Olga Ershova, Marina Ballyzek, Olga Smolenskaya, Sergey S. Yakushin, Dmitry Zateyshchikov, Mikhail Arkhipov, Alexandr Kuzmenko, Ivan Maksimov, Igor Motylev, Vladimir Rafalskiy, Leonid Strongin, Tatyana Treshkur, Natalya Volkova, Olga Barbarash, Tatiana Raskina, Leonid Bartosh, Inna Nikolskaya, Elena Shutemova, Viktor Gurevich, Natalia Burova, Elena Vorobyeva, Denis Andreev, Boris Bart, Tatiana Khlevchuk, Lyudmila Gapon, Ivan Gordeev, Nikolai Gratsiansky, Alsu Zalevskaya, Sergey Sayganov, Oleg Solovyev, Galina Reshedko, Natalia Shilkina, Petr Chizhov, Julia Shapovalova, Alexander Sherenkov, Olga Reshetko, Vladimir Simanenkov; **Spain:** Juan Garcia Puig, Jose Saban, Jose Pascual, Jose Dominguez, Elias Delgado, Carlos Calvo, Manuel Vida, Santiago Duran, Francisco Tinahones, Jordi Salas, Jose Miguel Gonzalez, Manuel Monreal, Armand Grau, Andreu Nubiola, Pere Alvarez; **Sweden:** Kaj Stenlöf, Pekka Koskinen, Carl-Johan

Lindholm, Ulrik Mathiesen, Katarina Berndtsson Blom, Bengt-Olov Tengmark, Hans Jul-Nielsen; **Ukraine:** Oleksandr Larin, Svetlana Panina, Svitlana Kovalenko, Olena Voloshyna, Vera Tseluyko, Olga Gyrina, Vadim Vizir, Olga Barna, Maryna Dolzhenko, Yuriy Mostovoy, Vadim Korpachev, Boris Mankovskiy, Mykola Vatutin; **United States:** Charles Arena, Basil Akpunonu, Rahfa Zerikly, Claire Baker, Toby Briskin, Darlene Bartilucci, Joshua Barzilay, Christian Breton, John Buse, Richard Cherlin, Michael Cobble, Clarence Ellis, Raymond Fink, Alan Forker, Ronald Garcia, Priscilla Hollander, Angela House, Daniel Hyman, Richard Ingebretsen, David Jack, Judith Kirstein, Kerri Kissell, Daniel Lorber, Donald McNeil, Wendell Miers, Alex Murray, Robert Call, Stephen T. Ong, Fernando Ovalle, Robert Pearlstein, Veronica Piziak, Daniel Pomposini, David Robertson, Julio Rosenstock, Ulrich Schubart, Shaukat Shah, Rodney Stout, Mark Turner, James Wallace, Leonard Chuck, Edmund Claxton, Emily Morawski, Alan Wynne, Carol Wysham, Michael Alderman, Walter Patton, Bryan Pogue, Arnold Silva, Roger Guthrie, Sam Lerman, Robert Madder, Wendy Miller, Daniel Weiss, Dean Kereiakes, Ronald J. Graf, Negah Rassouli, James Greenwald, Hanna Abu-Nassar, Derek Muse, Vicki Kalen, Natalia Hegedosh, Richard Dobrusin, Glover Johnson, Tami Bruce, Gary Gleason.

CANVAS-R

Argentina: Marisa Vico, Sonia Hermida, Lucrecia Nardone, Laura Maffei, Javier Farias, Elizabeth Gelersztein, Maximiliano Sicer, Andres Alvarisqueta, Georgina Sposetti, Virginia Visco, Rodolfo Feldman, Silvia Orio; **Australia:** Christopher Nolan, Michael Suranyi, Samantha Hocking, Stephen Stranks, Duncan Cooke, Ferdinandus de Looze, Ashim Sinha, Timothy Davis, Anthony Russell, Acharya Shamasunder, Murray Gerstman, Richard MacIsaac; **Belgium:** Chris Vercammen, Luc Van Gaal, Chantal Mathieu, Xavier Warling, Jan Behets, Andre Scheen, Guy T'Sjoen, Ann Verhaegen, Isabelle Dumont, Youri Taes, Francis Duyck, Fabienne Lienart; **Brazil:** Adolfo Sparenberg, Adriana Costa e Forti, Andressa Leitao, Cariolina Jungers di Siqueira Chrisman, César Hayashida, Daniel Panarotto, Fabio Rossi dos Sanos, Fadlo Fraige Filho, Flávia Coimbra Maia, Gilmar Reis, Hugo Lisboa, Joao Felicio, Joselita Siqueira, Lilia Nigro Maia, Luiz Alberto Andreotti Turatti, Maria José Cerqueira, Maria Tereza Zanella, Patricia Muszkat, Miguel Nasser Hissa, Teresa Bonansea; **Canada:** Igor Wilderman, Vincent Woo, Richard Dumas, Francois Blouin, Pierre Filteau, George Tsoukas, Peter Milne, Dan Dattani, Chantal Godin, Michael Omahony, Daniel Shu, Jasmin Belle-Isle, Douglas Friars, Anil Gupta, Ted Nemtean, Andrew Steele; **China:** Zhan-Quan Li, Changsheng Ma, Linong Ji, Shuguang Pang, Yan Jing, Ruiping Zhao, Ruifang Bu; **Czech Republic:** Tomas Spousta, Tatana Souckova, Dagmar Bartaskova, Pavlina Kyselova, Lea Raclavska, Milan Kvapil, Jana Havelkova, Emilia Malicherova; **France:** Philippe Zaoui, Didier Gouet, Jean-Pierre Courreges, Salha Fendri, Samy Hadjadj, Bruno Verges, Bogdan Nicolescu Catargi, Sylvaine Clavel, Jean-Jacques Altman, Agnes Hartemann, Gaétan Prevost; **Germany:** Diethelm Tschöpe, Elena Henkel, Rolf Göbel, Jochen Seufert, Hermann Haller, Thomas Behnke, Andreas Pfützner, Gerhard Klausmann, Klaus Busch, Baerbel Hirschhaeuser, Stephan Jacob; **Great Britain:** Melanie Davies, Rob Andrews, Narayan Annamalai, Hamish Courtney, Srikanth Bellary, Mark Blagden, John Clark, Steven Creely, Ken Darzy, Iskandar Idris, Richard Falk, Lucinda Summers, Njaimeh Asamoah, Andrew Johnson, See Kwok, Shenaz Ramtoola, Gerry Rayman, Jamie Smith, John Wilding; **Hungary:** Marietta Baranyai, Katalin Csomos, Mihaly Gurzo, Eleonóra Harcsa, Nikosz Kanakaridisz, Nóra Késmárki, Tamas Oroszlan, József Pátkay, Eva Peterfai, Balázs Gaszner, Ildiko Jozsef; **Italy:** Stefano Genovese, Antonio Ettore Pontiroli, Enzo Bonora, Dario Giugliano, Domenico Cucinotta, Giorgio Sesti, Paola Ponzani, Giuseppe Pugliese, Giulio Marchesini Reggiani, Paolo Pozzilli, Sergio Leotta, Emanuela Orsi, Carlo Giorda, Paolo Di Bartolo; **Korea:** Tae-Sun Park, Chung-Gu Cho, In-Joo Kim, Il Seong Nam-Goong, Choon Hee Chung, Ho Chan Cho, Dong-Seop Choi, Kun-Ho Yoon, Nan-Hee Kim, Kyung-Mook Choi, Kyu-Jeung Ahn, Ji-Oh Mok, Soon-Jib Yoo, Tae-Keun Oh, Kwan-Woo Lee, Hak-Chul Jang, Jeong-Hyun Park, In-Kyu Lee, Byung-Joon Kim, Doo-Man Kim, Ho Sang Shon, Moon-Kyu Lee, ShinGon Kim; **Malaysia:** Mafauzy Mohamed, Paranthaman Vengadasalam, Alexander Tong Boon Tan, Wan Mohd Izani Wan Mohamed, Rajesh P. Shah, Khalid Yusoff, Amir Sharifuddin Mohd Khir, Florence Tan, Mansor Yahya; **Mexico:** Rafael Violante, Manuel Odin De los Rios, Marco Alcocer, Enrique Morales, Juan Rosas, Armando Vargas, Manuel González, Esperanza Martinez, Jorge Antonio Aldrete, Guillermo Gonzalez, Cynthia Mustieles Rocha, Leobardo Sauque, Paul Frenk, José Luis Arenas; **The Netherlands:** Peter Tichelaar, A. Kooy, Albert Van de Wiel, Gerben Lochorn, Peter De Vries, Hans Feenstra, Max Nieuwdorp, Wouter Van Kempen, Mazin Alhakim, Ben Imholz, Ruud van Leendert, Peter Smak Gregoor, Joop Brussen, Hanno Pijl, Manuel Castro Cabezas, F. Gonkel, P. Smits, Daan Lansdorp, Susanne Kentgens, Aletha Veenendaal, Gloria Rojas; **New Zealand:** John Richmond, Russell Scott, Mike Williams, Dean Quinn, Jeremy Krebs, John Baker, Veronica Crawford, Calum Young; **Poland:** Malgorzata Arciszewska, Krystyna Jedynasty, Dariusz Sowinski, Ewa Szyprowska, Andrzej

Madej, Miroslawa Polaszewska-Muszynska, Danuta Zytkiewicz-Jaruga, Katarzyna Wasilewska, Piotr Romanczuk, Anna Ocicka-Kozakiewicz, Czeslaw Marcisz, Boguslaw Okopien, Anna Bochenek, Lukasz Wojnowski, Teresa Sliwinska, Barbara Rewerska, Witold Zmuda, Katarzyna Klodawska, Ewa Skokowska, Jacek Fabisiak, Cezary Danilkiewicz; **Puerto Rico:** Elba Perez Vargas, Elizabeth Barranco Santana; **Russia:** Tatiana Raskina, Olga Barbarash, Leonid Bartosh, Igor Motylev, A. Kuzin, Olga Reshetko, Tatyana Zykova, Olga Ershova, Marina Balyzek, Vladimir Rafalsky, Natalya Volkova, Nina Nosova, Natalia Burova, Alsu Zalevskaya, Galina Reshedko, Natalia Shilkina, Petr Chizhov, Alexander Sherenkov, Vladimir Simanenkov, Tatiana Lysenko, Irina Ipatko, Mikhail Boyarkin, Sergey Vorobyev, Lyudmila Gapon, Andrey Obrezan, Valeria Esip, Zhanna Paltsman, Andrey Verbovoy, Fatima Khetagurova, Yuri Shvarts; **Spain:** Pere Alvarez-Garcia, Francisco Martinez Deben, Josep M. Grinyo, Carlos Calvo, Carmen Suarez, J.M. Pascual, Jose Dominguez, Anna Oliveras, Armand Grau, Fernando Gómez Peralta, Luis Alvarez-Sala, Cañizo Francisco, Jorge Gómez Cerezo, Juan Garcia Puig, Carlos Trescolí, Francisco Jose Fuentes Jimenez, Santiago Tofé, Judith López, Javier Nieto Iglesias, Luis Vigil, Santiago Duran Garcia, Jose Luis Gorriz, Pilar Saavedra Vallejo, Francisco Tinahones Madueno, Jose Luis Blanco Coronado, Alfonso Soto, Luis De Teresa, Jose Miguel Gonzalez, Antonio Rodriguez Botaro, Carmina Cuesta; **Sweden:** Bjorn Bragée, Bengt-Olov Tengmark, Hans Jul-Nielsen, Pekka Koskinen, Linda Moris, Fredrik Huss, Pär Jennersjö, Katarina Berndtsson-Blom, Bo Liu, Kaj Stenlöf, Carl-Johan Lindholm, Johan Jendle; **Taiwan:** Dee Pei, Wayne H.-H. Shue, Chern-En Chiang, Ching-Chu Chen, Ming-Nan Chien, Ping-Yen Liu, Ching-Ling Lin, Yi-Jing Sheen; **Ukraine:** Dmytro Reshotko, Nikolay Rishko, Olexander Samoylov, Valentina Serkova, Ivan Smirnov, Liubov Sokolova, Vira Tseluyko, Vadym Vizir, Tetiana Zlova, Vitaliy Maslyanko, Oleksandr Larin, Valentina Velichko, Lyudmila Prystupa, Nadiya Yarema, Galina Mishanich, Iryna Bondarets, Nataliya Virstyuk, Olexander Serhiyenko, Stepan Pavlyk, Olena Levchenko, Orest Abrahamovych, Volodymyr Botsurko, Maryna Dolzhenko, Victoria Chernikova, Yuriy Karachentsev, Vitaliy Katerenchuk, Vadym Korpachov, Yaroslav Malynovsky, Boris Mankovsky, Yuriy Mostovoy, Larisa Pererva, Nataliya Pertseva; **United States:** Vicki Conrad, Kenneth Fox, David Jack, Robert Buynak, Michael Dever, John Kirby, Larry Odekirk, Priyantha Wijewardane, Robert Carson, Bruce Seaton, Ann Elizabeth Mohart, Salvatore Bianco, Michael R. Cox, Andrew Kim, Steven Geller, Jakkidi Reddy, Derek Muse, Alan Wynne, Harold Bays, Judith Kirstein, James Riser, Ahmed Arif, Claire Baker, Kim Barbel-Johnson, Gary Bedel, Pierre Blemur, Christian Breton, Anna Chang, Brian Naccari, Nancy Jo Coburn, Lisa Cohen, Eric Dedeke, Charles Diederich, John Earl, Anu George, Matthew Gilbert, Gary Gleason, Gregory Haase, Rodney Ison, Mahendra Jain, Imtiaz Alam, Sam Lerman, Lawrence Levinson, Lon D. Lynn, Michael Oliver, Barry Kusnick, Robert Pearlstein, Sanford Plevin, Samuel Mujica Trenche, Vernon Young, Michael Jutovsky, Ralph Wade, James Wallace, Albert Weisbrot, Duane Wombolt, Alan Forker, Jalal Taslimi, Roger Guthrie.
